# Training opportunities for noncommunicable diseases research in Latin America: A scoping review

**DOI:** 10.26633/RPSP.2019.68

**Published:** 2019-08-22

**Authors:** Jessica Hanae Zafra-Tanaka, Rodrigo M. Carrillo-Larco, Antonio Bernabe-Ortiz, Vilma Edit Irazola, Goodarz Danaei, J. Jaime Miranda

**Affiliations:** 1 CRONICAS Centre of Excellence in Chronic Diseases CRONICAS Centre of Excellence in Chronic Diseases Universidad Peruana Cayetano Heredia Lima Peru CRONICAS Centre of Excellence in Chronic Diseases, Universidad Peruana Cayetano Heredia, Lima, Peru.; 2 Department of Epidemiology and Biostatistics Department of Epidemiology and Biostatistics School of Public Health, Imperial College London London United Kingdom Department of Epidemiology and Biostatistics, School of Public Health, Imperial College London, London, United Kingdom.; 3 South American Center of Excellence for Cardiovascular Health South American Center of Excellence for Cardiovascular Health Institute for Clinical Effectiveness and Health Policy Buenos Aires Argentina South American Center of Excellence for Cardiovascular Health, Institute for Clinical Effectiveness and Health Policy, Buenos Aires, Argentina.; 4 Department of Global Health and Department of Epidemiology Department of Global Health and Department of Epidemiology Harvard T. H. Chan School of Public Health, Boston Massachusetts United States of America Department of Global Health and Department of Epidemiology, Harvard T. H. Chan School of Public Health, Boston, Massachusetts, United States of America.

**Keywords:** Noncommunicable diseases, research, education, research personnel, supply & distribution, Latin America, Enfermedades no transmisibles, investigación, educación, investigadores, provisión & distribución, América Latina, Doenças não transmissíveis, educação, pesquisa, pesquisadores, provisão & distribução, America Latina

## Abstract

**Objective.:**

To identify gaps in postgraduate training and options for building capacity in noncommunicable disease (NCDs) research in Latin America.

**Methods.:**

This was a scoping review of postgraduate opportunities in NCDs at top universities in Latin America and of training grants awarded by international funding bodies. Three global university rankings were considered—the QS Ranking, the Shanghai Ranking, and the Times Ranking. Latin American universities appearing in at least two of these were selected. University websites were searched for current graduate programs in biostatistics, epidemiology, global health, health economics, and public health. Information was extracted, summarized, and evaluated to identify any programs focused on NCDs. In addition, seven international funding bodies’ websites were searched for training grants.

**Results.:**

In all, 33 universities offering 72 postgraduate programs met the inclusion criteria. One of these programs was exclusively devoted to NCD, and 12 offered NCDs as a dissertation research topic. Only two training grants were awarded to a Latin American institution for NCD capacity building. There are few NCD research training programs in Latin America and only one program exclusively focused on NCDs.

**Conclusion.:**

There seem to be few NCD-specific research training programs in Latin America. Leveraging existing programs and expanding those with a focus on NCDs could help enhance NCD research capacity in the region. These initiatives should be supported by international funding agencies through more funding opportunities.

## RESUMEN

**Objetivo.:**

Detectar las brechas en la formación a nivel de posgrado y las opciones disponibles para fortalecer las capacidades de investigación sobre enfermedades no transmisibles (ENT) en América Latina.

**Métodos.:**

Se realizó una revisión exploratoria de los programas de posgrado sobre las ENT disponibles en las mejores universidades de América Latina y de las becas para investigadores otorgadas por los organismos internacionales de financiamiento. Se consideraron tres listas de clasificación académica de universidades del mundo: QS Ranking, Shanghai Ranking y Times Ranking. Se seleccionaron las universidades de América Latina que figuraban en al menos dos de ellas. Se utilizaron los sitios web de las universidades para buscar los programas de posgrado actuales en las áreas de bioestadística, epidemiología, salud mundial, economía de la salud y salud pública. La información se extrajo, se resumió y se evaluó para encontrar todos los programas centrados en las ENT. Además, se realizó una búsqueda de las becas de formación ofrecidas en los sitios web de siete organismos internacionales de financiamiento.

**Resultados.:**

En total, 33 universidades que ofrecían 72 programas de posgrado reunieron los criterios de inclusión. Uno de estos programas estaba exclusivamente dedicado a las ENT, y doce incluían las ENT como tema de investigación en tesis de grado. Solo dos becas de formación fueron otorgadas a una institución latinoamericana para fortalecer las capacidades de investigación sobre las ENT. Existen pocos programas para la formación de investigadores sobre las ENT en América Latina y hay un solo programa exclusivamente centrado en las ENT.

**Conclusiones.:**

Al parecer, hay pocos programas específicos para la formación de investigadores sobre las ENT en América Latina. Aprovechar mejor los programas existentes y ampliar aquellos que se centran en las ENT podría ayudar a aumentar las capacidades de investigación sobre ENT en la región. Estas iniciativas deben contar con el apoyo de los organismos internacionales de financiamiento mediante el aumento de las oportunidades de financiamiento.

## RESUMO

**Objetivo.:**

Identificar lacunas no ensino de pós-graduação e cursos de formação em pesquisa em doenças não transmissíveis na América Latina.

**Métodos.:**

Trata-se de uma revisão da literatura do tipo scoping review das oportunidades de pós-graduação em doenças não transmissíveis nas principais universidades da América Latina e das bolsas de estudo oferecidas por organismos internacionais de financiamento. O estudo se baseou em três rankings mundiais de universidades – QS Ranking, Ranking de Xangai e The Times Higher Education World University Ranking – e as universidades latino-americanas que figuravam em pelo menos dois destes rankings foram incluídas na análise. Nos sites das universidades, foi feito um levantamento dos cursos atuais de pós-graduação em bioestatística, epidemiologia, saúde global, economia da saúde e saúde pública. As informações obtidas foram sumarizadas e avaliadas para identificar os cursos em doenças não transmissíveis. Além disso, nos sites de sete organismos internacionais de financiamento, foi feita uma pesquisa das bolsas de estudo oferecidas.

**Resultados.:**

Ao todo, 33 universidades com 72 cursos de pós-graduação atenderam os critérios de inclusão no estudo. Verificou-se que um curso tinha foco exclusivo no estudo de doenças não transmissíveis e 12 cursos tinham doenças não transmissíveis como tópico de pesquisa de dissertação. Foram concedidas somente duas bolsas de estudo em doenças não transmissíveis a uma instituição latino-americana. Observou-se um pequeno número de programas de formação em pesquisa de doenças não transmissíveis na América Latina, com apenas um programa com foco exclusivo nestas doenças.

**Conclusões.:**

Existem poucos programas de formação em pesquisa especificamente dedicados ao estudo de doenças não transmissíveis na América Latina. Faz-se necessário tirar proveito dos programas existentes e ampliar os programas com foco em doenças não transmissíveis para melhorar a capacidade de pesquisa em doenças não transmissíveis na Região. Essas iniciativas devem receber o apoio de organismos internacionais com maior oferta de bolsas de estudo.

Noncommunicable diseases (NCDs) account for more than one-half of the global disability-adjusted life years (DALYs), and represent more than three-quarters of DALYs in Latin America (1). Several institutions have called attention to the need for developing research capacity in NCDs to combat the economic impact of the NCD pandemic (1).

Research training programs—formal academic programs in areas such as epidemiology and public health—are important for building capacity. These programs allow professionals to develop the skills necessary for conducting high-quality research. A successful example is a clinical research and capacity building program for NCDs prevention and control held in India since 2001; it has now trained more than 2 000 health professionals who have been granted over 30 projects by various international institutions (2). Another successful program is the International Tobacco and Health Research and Capacity Building Program, which aims to promote research and train professionals to reduce tobacco burden. Started in 2002, it has now trained more than 3 000 professionals in low- and middle-income countries around the world and facilitated international collaborations to develop their research capacity (3).

Only a few education institutions in Latin America focus on research in NCDs (4, 5) and their training capacity does not meet current needs. Furthermore, in countries where the available training opportunities may be sufficient, they fall short on the regional level where comprehensive research training is needed to implement evidence-based interventions.

This scoping review sought to identify training gaps and options for building NCD research capacity in Latin America by summarizing and synthesizing the currently-available training programs and grants in the region.

## MATERIALS AND METHODS

### Study design

This was a scoping review of training opportunities in NCD research at top universities in Latin America and training grants awarded by international funding bodies. Training opportunities were defined as formal academic programs in NCD related areas, i.e., biostatistics, epidemiology, global health, health economics, and public health.

Scoping review methodology was chosen because it “comprehensively summarizes and synthesizes evidence with the aim of informing practice, programs, and policy, providing direction to future research priorities” (6). In this context, the study sought to identify and describe training opportunities that could inform new programs and educational policies, thereby potentially building capacity in the region. By doing so, two goals were attained: (i) an estimate of the number of academic institutions offering graduate research programs with a concentration in NCD and an understanding of their key characteristics; and (ii) an estimate of the number of research training grants awarded by international funding bodies through May 2018.

### Data sources

#### Available postgraduate programs.

Three university rankings, the QS ranking (7), the Shanghai ranking (8), and the Times ranking (9), were chosen for determining the top universities in Latin America in 2017. Those universities that were listed within the top 500 universities in the world in at least two of the three rankings, regardless of their ranking position, were selected for inclusion. Two independent researchers evaluated each institution’s official website, identifying all postgraduate (master’s and doctoral) programs on biostatistics, epidemiology, global health, health economics, and public health. An information extraction form was developed and used to summarize key features (see Variables section) of each program (e.g., length of program, core courses, costs/funding). When these details were unavailable or unclear, the researchers emailed the contact person to request the information. The search and email inquiries were conducted in February — May 2018.

#### Research training grants.

Relevant grant opportunities offered by key global funding agencies were reviewed. These key agencies were: Grand Challenges Canada (10), International Development Research Centre from Canada (IDRC; 11), the United States National Institutes of Health (NIH; 12), Swiss National Science Foundation (13), Swiss Agency for Development and Cooperation (14), the EU Framework Programme for Research and Innovation (15), and the Wellcome Trust (16). These organizations were chosen because of their history of successful interactions with research groups in Latin America.

The searches were conducted in March – April 2018, except for the Research Portfolio Online Reporting Tools (RePORT) which was searched in February 2018. Each search was modified according to the agency and its available information, specifically:

- Grand Challenges Canada—innovations within priority areas, i.e., cancer cardiovascular diseases, chronic respiratory diseases, dementia, depression, diabetes, epilepsy, hypertension, and noncommunicable diseases.
- IDRC— stories (successful applicants) with the topic *health*.
- NIH—specific grants, International Research Training Planning Grants (code D71) and International Research Training Grants (code D43), available from the Research Portfolio Online Reporting Tools (17) (18). No fiscal year restrictions were applied.
- Swiss Agency for Development and Cooperation*—*planned, active, and completed projects with *health as* the topic and *noncommunicable diseases* as the subtopic available from the projects database (https://www.eda.admin.ch/deza/en/home/activities-projects/projekte-fokus/Project-database.html).
- Swiss National Science Foundation—grants awarded to programs focused on international cooperation, including searches of the Swiss Programme for Research on Global Issues for Development (19) and the country’s bilateral programs with Brazil (20) and Argentina (21).
- EU Framework Programme for Research and Innovation (HORIZON 2020) projects on *health* (https://sme.easme-web.eu/#) (22).
- Wellcome Trust—grants awarded from 2005 – 2017 (https://wellcome.ac.uk/funding/people-and-projects/grant-funding-data) and training grants awarded to researchers in Latin America (16).

### Variables

The characteristics examined for each program were: (i) focus on NCD (yes/no if stated in the aim, introduction, or presentation of the program); (ii) length of the program (years proposed for program completion/degree); (iii) modality (face-to-face or online); (iv) frequency (days per week of required class/online attendance); (v) prerequisites and requirements for admission and completion; and (iv) total cost of the program (in US dollar except public universities in Brazil which are free of charge by law). Each program’s objectives and curriculum were also retrieved.

### Statistical analysis

All the aforementioned variables and data were collected using a Microsoft Excel™ (Microsoft Corp., Redmond, Washington, United States) spreadsheet. Statistical analysis was performed using Excel™ software. The unit of analysis was one program or training grant awarded. Absolute and relative frequencies were used to describe categorical variables, while central tendency and dispersion measures were used for quantitative variables

### Ethics

This study did not require IRB approval as human subjects did not participate.

## RESULTS

### Available postgraduate programs

Regarding the QS ranking, of the top 500 universities for medicine, 35 were in Latin America. With respect to the Shanghai ranking, of the top 500 universities for public health, 20 were in Latin America. Finally, of the top 596 universities for medicine or dentistry in the Times ranking, 59 were in Latin America. A total of 33 universities were included in at least two of the three rankings (Table 1) and were located in seven countries: Brazil (*n* = 17), Chile (*n* = 5), Colombia (*n* = 5), Mexico (*n* = 3), Argentina (*n* = 1), Peru (*n* = 1), and Venezuela (*n* = 1).

**TABLE 1.** Latin American universities listed in at least two of three international rankings—QS Ranking, the Shanghai Ranking, and the Times Ranking, 2017

University	Country
Instituto Tecnológico y de Estudios Superiores de Monterrey	Mexico
Pontificia Universidad Católica de Chile	Chile
Pontificia Universidad Javeriana	Colombia
Pontificia Universidade Católica do Rio Grande do Sul	Brazil
Universidad Austral de Chile	Chile
Universidad Central de Venezuela	Venezuela
Universidad de Antioquia	Colombia
Universidad de Buenos Aires	Argentina
Universidad de Chile	Chile
Universidad de Concepción	Chile
Universidad de Guadalajara	Mexico
Universidad de los Andes	Colombia
Universidad de Santiago de Chile	Chile
Universidad del Rosario	Colombia
Universidad Nacional Autónoma de México	Mexico
Universidad Nacional de Colombia	Colombia
Universidad Peruana Cayetano Heredia	Peru
Universidade de Brasília	Brazil
Universidade do Estado do Rio de Janeiro	Brazil
Universidade Estadual de Campinas	Brazil
Universidade Estadual Paulista	Brazil
Universidade Federal da Bahia	Brazil
Universidade Federal de Goiás	Brazil
Universidade Federal de Minas Gerais	Brazil
Universidade Federal de Pelotas	Brazil
Universidade Federal de Pernambuco	Brazil
Universidade Federal de Santa Catarina	Brazil
Universidade Federal de São Paulo	Brazil
Universidade Federal do Ceará	Brazil
Universidade Federal do Paraná	Brazil
Universidade Federal do Rio de Janeiro	Brazil
Universidade Federal do Rio Grande Do Sul	Brazil
University of São Paulo	Brazil

**FIGURE 1.**
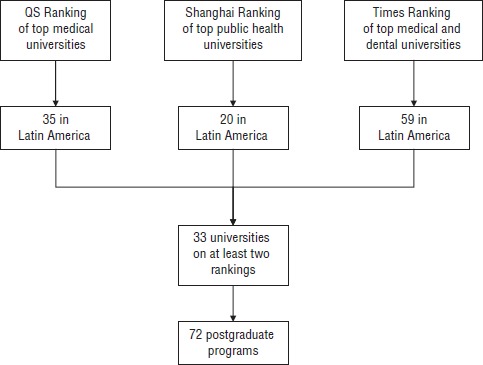
Flowchart of the process for selecting the Latin American universities to be included in a study of training programs in noncommunicable diseases research

***Source:*** Prepared by authors from the study results.

A total of 72 biostatistics, epidemiology, global health, health economics, and public health postgraduate programs were found to be offered by the universities for which information was accessible (Figure 1).

### Characteristics of the postgraduate programs

One-half of the programs found were located in Brazil. Most of the programs found were MPH (36.1%) or PhD (36.1%); the remainder were MSc (27.8%). All of the programs were face-to-face. Most of the master’s programs had a duration of 2 years (40/43), ranging from 1 – 3 years with some programs allowing up to 4 years to graduate. Most of the doctoral programs had a duration of 4 years (21/25), ranging from 3 – 5 years, with some allowing up to 8 years to graduate (Tables 2 and 3).

Only 61 postgraduate programs reported their requisites for graduation. All required a dissertation, with one having an option to complete an unspecified equivalent, and two requiring a dissertation plus practice as a teaching assistant.

Schedules varied widely across programs. Almost one-third required 2 – 5 days of classes per week (16/59). Others permitted students to design their own schedules (37/59). Only six programs required full time or 6 days per week.

Of the 72 programs, only one was devoted to health economics and one to NCD—these programs explicitly stated that NCD was their principle topic. The latter was a clinical research program focused on hypertension. In addition, 12 of 72 had NCDs as a suggested research topic. These 12 programs had an institute or a senior researcher in charge of guiding students interested in NCD research.

### Research training grants

There were no past funding opportunities devoted to developing NCD training programs in Latin America among the grants awarded by Grand Challenges Canada, IDRC, the Swiss National Science Foundation, the Swiss Agency for Development and Cooperation, the EU Framework Programme for Research and Innovation, and the Wellcome Trust.

**TABLE 2.** Characteristics of postgraduate programs in biostatistics, epidemiology, global health, health economics, and public health offered by Latin American universities, 2018

Program	University	Country	Degree	Duration (years)	Website
Doutorado em Epidemiologia	Universidade de São Paulo	Brazil	PhD	4	https://bit.ly/326bc3m
Mestrado em Saúde Pública	Universidade de São Paulo	Brazil	MPH	2	https://bit.ly/2XqXMLU
Doutorado em Saúde Pública	Universidade de São Paulo	Brazil	PhD	4	https://bit.ly/2XqXMLU
Doutorado em Saúde Global e Sustentabilidade	Universidade de São Paulo	Brazil	PhD	4	https://bit.ly/2LzAoJK
Magíster en Epidemiología	Pontificia Universidad Católica de Chile	Chile	MSc	1.5	https://bit.ly/32a2HUU
Magister en Salud Pública	Pontificia Universidad Católica de Chile	Chile	MPH	1.5	https://bit.ly/2e5LmG7
Magíster en Bioestadística	Universidad de Chile	Chile	MSc	2	https://bit.ly/2Kvh0KV
Magíster en Salud Pública	Universidad de Chile	Chile	MPH	2	https://bit.ly/2HRqlLg
Doctorado en Salud Pública	Universidad de Chile	Chile	PhD	4	https://bit.ly/2FGegqw
Mestrado em Saúde Coletiva	Universidade Federal de São Paulo	Brazil	MPH	2	https://bit.ly/2JPATLx
Doutorado em Saúde Coletiva	Universidade Federal de São Paulo	Brazil	PhD		https://bit.ly/2JPATLx
Mestrado em Saúde Pública	Universiade Federal de Minas Gerais	Brazil	MPH	2	https://bit.ly/2JNu9xG
Doutorado em Saúde Pública	Universiade Federal de Minas Gerais	Brazil	PhD	4	https://bit.ly/2JNu9xG
Mestrado em Epidemiologia	Universidade Federal do Rio Grande Do Sul	Brazil	MSc	2	https://bit.ly/2jt3j36
Doutorado em Epidemiologia	Universidade Federal do Rio Grande Do Sul	Brazil	PhD	4	https://bit.ly/2FQoyHC
Mestrado em Saúde Coletiva	Universidade Federal do Rio Grande Do Sul	Brazil	MPH	2	https://bit.ly/2rjoyso
Maestría en Bioestadística	Pontificia Universidad Javeriana	Colombia	MSc	2	https://bit.ly/2HRQrOl
Maestría en Epidemiología clínica	Pontificia Universidad Javeriana	Colombia	MSc	3	https://bit.ly/2rkAp8R
Maestría en Salud Pública	Pontificia Universidad Javeriana	Colombia	MPH	2	https://bit.ly/2wdlPWl
Doctorado en Epidemiología Clínica	Pontificia Universidad Javeriana	Colombia	PhD	5	https://bit.ly/2Kyvjyo
Doctorado en Salud Pública - Bogotá	Universidad Nacional de Colombia	Colombia	PhD	4	https://bit.ly/2FGD5Cw
Maestría en Salud Pública - Bogotá	Universidad Nacional de Colombia	Colombia	MPH	2	https://bit.ly/2HPh3nl
Maestría en Epidemiología clínica - Bogotá	Universidad Nacional de Colombia	Colombia	MSc	2	https://bit.ly/2rkrzbU
Doctorado en Epidemiología - Medellín	Universidad de Antioquia	Colombia	PhD	4	https://bit.ly/2rk66Qt
Doctorado en Salud Pública - Medellín	Universidad de Antioquia	Colombia	PhD	4	https://bit.ly/2wbGdaf
Maestría en Epidemiología - Medellín	Universidad de Antioquia	Colombia	MSc	2	https://bit.ly/2w6xw0O
Maestría en Salud Pública - Medellín	Universidad de Antioquia	Colombia	MPH	2	
Mestrado em Saúde Coletiva	Universidade Federal de Santa Catarina	Brazil	MPH	2	https://bit.ly/2xunDb9
Doutorado em Saúde Coletiva	Universidade Federal de Santa Catarina	Brazil	PhD	4	https://bit.ly/2xunDb9
Mestrado em Saúde Coletiva	Universidad Federal Rio de Janeiro	Brazil	MPH	2	https://bit.ly/2rfOKDk
Doutorado em Saúde Coletiva	Universidad Federal Rio de Janeiro	Brazil	PhD	2	https://bit.ly/2rjhET2
Mestrado em Saúde Coletiva	Universidad de Brasilia	Brazil	MPH	2	https://bit.ly/2XrJOtj
Doutorado em Saúde Coletiva	Universidad de Brasilia	Brazil	PhD	4	https://bit.ly/2FSeldI
Maestría en salud pública	Universidad de Buenos Aires	Argentina	MPH	2	https://bit.ly/2RRtIId
Maestría en investigación en ciencias médicas	Universidad de Buenos Aires	Argentina	MSc	2	https://bit.ly/2LANaHT
Mestrado em Saúde Coletiva	Universidade Estadual Paulista	Brazil	MPH	2	https://bit.ly/2xsmTmL
Doutorado em Saúde Coletiva	Universidade Estadual Paulista	Brazil	PhD	4	https://bit.ly/2xsmTmL
Mestrado Profissional em Rede de Saúde Coletiva	Universidade Estadual Paulista	Brazil	MPH	2	https://bit.ly/2L0o2Ll
Mestrado Profissional de pesquisa clinica	Universidade Estadual Paulista	Brazil	MSc	2	https://bit.ly/2XrX8mh
Maestría en Epidemiología Clínica	Universidad Peruana Cayetano Heredia	Peru	MSc	2	https://bit.ly/2Ywgras
Maestría en Salud Pública y Salud Global	Universidad Peruana Cayetano Heredia	Peru	MPH	2	https://bit.ly/2S2CX8B
Maestría en Ciencias en Investigación Epidemiológica	Universidad Peruana Cayetano Heredia	Peru	MSc	1	https://bit.ly/2L25bPY
Maestría en Informática Biomédica en Salud Global	Universidad Peruana Cayetano Heredia	Peru	MSc	2	https://bit.ly/2L25sT0
Doctorado en Ciencias en Investigación Epidemiológica	Universidad Peruana Cayetano Heredia	Peru	PhD	3	https://bit.ly/2FS3EI5
Doctorado en Salud Pública	Universidad Peruana Cayetano Heredia	Peru	PhD	3	https://bit.ly/2xwlA6o
Maestría en Salud Pública	Universidad de Guadalajara	Mexico	MPH	2	https://bit.ly/2JdbI8p
Maestría en investigación clínica	Universidad de Guadalajara	Mexico	MSc	2	https://bit.ly/2RRtS2h
Doctorado en Salud Pública	Universidad de Guadalajara	Mexico	PhD	4	http://bit.ly/2S2DjMt
Doctorado en investigación clínica	Universidad de Guadalajara	Mexico	PhD	4	http://bit.ly/2NACFHa
Maestría en Salud Pública	Universidad de los Andes	Colombia	MPH	2	http://bit.ly/2KY76Vw
Maestría en Epidemiología	Universidad de los Andes	Colombia	MSc	2	http://bit.ly/2RUGcik
Mestrado Academico em Saudé Colectiva	Universidade do Estado do Rio de Janeiro	Brazil	MPH	2	http://bit.ly/2RQpfp2
Doutorado em Saúde Coletiva	Universidade do Estado do Rio de Janeiro	Brazil	PhD	4	http://bit.ly/2RQpfp2
Mestrado em Saúde Coletiva	Universidade Federal da Bahia	Brazil	MPH	2	http://bit.ly/2xx0tAR
Doutorado em Saúde Coletiva	Universidade Federal da Bahia	Brazil	PhD	5	http://bit.ly/2FSBXP8
Mestrado Profissional em Saúde Coletiva	Universidade Federal da Bahia	Brazil	MPH	2	http://bit.ly/2KYGUKx
Mestrado em Saúde Coletiva	Universidade Federal do Paraná	Brazil	MSc	2	http://bit.ly/2XnYaL1
Mestrado em Epidemiologia	Federal University of Pelotas	Brazil	MSc		http://bit.ly/2LDK06c
Epidemiología de las Enfermedades Endémicas	Universidad Central de Venezuela	Venezuela	MSc		http://bit.ly/2XLHXna
Maestría en Investigación Clínica	Universidad Central de Venezuela	Venezuela	MSc		http://bit.ly/2RX9IUn
Doctorado en Salud Pública	Universidad Central de Venezuela	Venezuela	PhD	4	http://bit.ly/2XPDe3G
Maestría en epidemiología	Universidad del Rosario	Colombia	MSc	2	http://bit.ly/2FSwR5E
Maestría en Salud Pública	Universidad del Rosario	Colombia	MPH	2	http://bit.ly/2xtXZ6r
Mestrado em Medicina Tropical e Saúde Pública	Universidade Federal de Goiás	Brazil	MSc	2	http://bit.ly/2JjznCU
Doutorado em Medicina Tropical e Saúde Pública	Universidade Federal de Goiás	Brazil	PhD	4	http://bit.ly/2JjznCU
Mestrado em Saúde Coletiva	Universidade Federal de Goiás	Brazil	MPH	2	http://bit.ly/30a8yHZ
Mestrado em Saúde Pública	Universidade Federal do Ceará	Brazil	MPH	2	http://bit.ly/2LCL4HB
Doutorado em Saúde Pública/Coletiva	Universidade Federal do Ceará	Brazil	PhD	4	http://bit.ly/2JoRHKY
Mestrado em Saúde Coletiva	Universidade Federal de Pernambuco	Brazil	MPH	2	http://bit.ly/2XEoxAL
Doutorado em Saúde Coletiva	Universidade Federal de Pernambuco	Brazil	PhD	4	http://bit.ly/2XEoxAL
Mestrado em Saúde Coletiva	Universidade Estadual de Campinas	Brazil	MPH	2	http://bit.ly/2XroatS
Doutorado em Saúde Coletiva	Universidade Estadual de Campinas	Brazil	PhD	4	http://bit.ly/2XroatS

**TABLE 3.** Summary of the characteristics of postgraduate programs offered by Latin American universities 2018

Characteristics	n	%
Country		
Argentina	2	2.7
Brazil	37	51.4
Chile	5	6.9
Colombia	15	20.8
Mexico	4	5.6
Peru	5	8.3
Venezuela	3	4.2
Academic degree		
MPH	26	36.1
MSc	20	27.8
PhD	26	36.1
Academic area		
Biostatistics	5	6.9
Epidemiology	24	33.3
Global health	2	2.8
Health economics	1	1.4
Noncommunicable diseases	1	1.4
Public health	49	68.1
Duration^a^	2 years	2 – 4 years
Tuition free of charge	37	58.7
Tuition per semester^a^	US$ 2 545	US$ 2 056 – 3 650

a Median (interquartile range). Does not total 72 when information was not available for all programs.

A total of 12 active International Research Training Planning Grants (D71) were available using the RePORT from NIH, of which 5 focused on NCDs (23-25) and the remaining on infectious diseases. Of the 5 NCD-oriented programs, only 1 was based in Peru. There were also 159 International Research Training Grants (D43) found using RePORT. Of these, 34 were devoted to NCDs; the remainder focused on infectious diseases or injuries. Of the grants dedicated to either a specific NCD or NCDs in general, two were awarded in Latin America: the “Interdisciplinary Cerebrovascular Diseases Training Program in South America,” from the University of Washington (Seattle, Washington, United States), *Universidad Peruana Cayetano Heredia* (Lima, Peru), and the *Instituto Nacional de Ciencias Neurológicas del Perú* (Lima, Peru) (5)*;* and “Promoting Capacity Building in Chronic Diseases Research in South America” from *Instituto de Efectividad Clínica y Sanitaria* (Buenos Aires, Argentina) and Harvard University (Cambridge, Massachusetts, United States) (4).

## DISCUSSION

### Main results

We found a total of 72 postgraduate training programs on biostatistics, epidemiology, global health, health economics, or public health offered by 33 universities in Latin America. The sole program devoted to NCDs was a master’s degree on clinical epidemiology offered in Venezuela. We also found 12 programs with NCDs as the research area—seven PhDs and five master’s—most of which were offered by universities in Brazil. We found only two training grants dedicated to NCDs, of which only one was a training planning grant; both were funded by the Fogarty International Center at the NIH. The limited opportunities for postgraduate training in NCD research signal the need to reshape, enhance, and update existing programs and develop new ones to address the shortage.

### Opportunities and challenges of available programs

We found only one available postgraduate program exclusively devoted to a noncommunicable disease, hypertension. It was based in Venezuela. However, there were several biostatistics, epidemiology, and public health programs that could be complemented with NCD research training, especially where there was an NCD senior researcher or research group in charge. Students would be able to develop methodological skills and possibly pursue a career in NCDs, though a research career would be better built on a formal training program, MSc or PhD, specifically focused on NCDs. Such formal NCD programs would improve the probability of adequate capacity-building and ensure a sufficient workforce of professionals dedicated to NCD research in Latin America.

Supporting early career investigators has been proposed as an action to move forward on the fight against NCDs. Creating an adequate training environment can be challenging because there is a dearth of researchers who can dedicate time to training, teaching, and mentoring activities (26, 27). This is indeed an important barrier to improved capacity building, though grants for these pursuits could encourage researchers to allocate time to training, teaching, and mentoring.

Regarding funding opportunities, to the best of our knowledge, only the Fogarty International Center at NIH offered training grants. However, most of the awarded proposals were oriented toward infectious diseases. This does not necessarily mean that applications were only aimed at communicable diseases. However, communicable diseases seem to make a stronger case for research funding than do NCDs, although the latter are responsible for more deaths worldwide. Or it could be that certain conditions have prompted a sharp rise in training and research funding for communicable disease; for example, the impact of climate on vector distribution. Such aspects need to be identified for NCD in Latin America; for example, high attitude locations could offer an interesting scenario for the study of NCDs under the physiological challenges of hypoxia (28).

All in all, training grants offered by funding institutions, such as Fogarty International Center and the Wellcome Trust, provide support to researchers for advancing in their careers (29, 30) and capabilities. And it is time for Latin America to expand and train the next cadre of NCDs-specific researchers. The robust methodology of this scoping review allowed us to draw convincing conclusions about the lack of training programs in Latin America, especially for NCDs research. Consequently, we implore research groups and universities in Latin America to apply to funding calls on training activities, and focus these on NCDs. Not long ago, high-quality training on infectious diseases was urgently needed. Response efforts led to several renowned institutions dedicated to research on infectious diseases—the *Instituto Nacional de Medicina Tropical* (31)** in Argentina and the *Instituto de Medicina Tropical Alexander von Humboldt* in Peru (32) are two such examples. Also, training programs were developed, such as the Gorgas Program in Peru and Panama (33, 34). Now, the same efforts are needed for NCDs.

### Limitations

As a scoping review, the study was dependent on response information being available for each review question (35). Thus, choosing programs offered only at universities listed on two or more of the chosen rankings could have introduced selection bias. That said, we believe that a junior researcher interested in pursuing a master’s or PhD program would likely look at top universities, i.e., those included in these rankings. Still, some universities lacked or had outdated information on their websites. When contacted, these institutions explained that their programs were not currently recruiting; therefore, it is possible that these were missed. However, even if all universities with missing information had an NCD-oriented training program, there would still be few, upholding the overall study findings and concerns.

Regarding training grants, most of the funding agencies did not have available records of past grants from funding calls. However, we minimized the chance of missing any record by performing a structured search on each agency’s website. It is unlikely that any training grant was missed because these agencies mostly fund research activities, with or without some training component, rather than training projects exclusively.

Regarding the Swiss Confederation’s bilateral programs with Brazil (15) and Argentina (16), specific details for each program were not accessible, so these results could not be generalized for this agency.

### Conclusions

There are few NCD-specific research training programs in Latin America, and only one exclusively focused on NCDs. Masters and PhD program directors could prioritize NCDs as a research theme within existing biostatistics, epidemiology, global health, health economics, and public health programs. This would allow newer researchers to work on NCD-related projects and develop skills and expertise on a specific NCD-related topic.

International funding agencies should respond to the increasing burden of NCDs in Latin America and provide more funding opportunities to implement NCDs training programs.

In spite of the scarce training opportunities found, we believe that leveraging from and expanding upon existing programs with an NCD focus within a larger theme could be valuable to enhanced NCD research capacity in the region. These initiatives should be supported by international funding agencies through more grant opportunities.

#### Author contributions.

All authors conceived the original idea. JHZT collected the data, JHZT and RCL analyzed the data, and all authors interpreted the results and wrote the paper. All authors reviewed and approved the final version.

#### Acknowledgement.

We thank Victoria Cavero for helping us communicate in Portuguese with the Brazilian Universities to ask for important information regarding the postgraduate programs.

#### Funding.

This project was founded by the Fogarty International Center National Institutes of Health, grant number 1D71TW010877-01. Rodrigo M. Carrillo-Larco was supported by a Wellcome Trust International Training Fellowship (214185/Z/18/Z). The funders had no role in the study design, data collection or analysis, decision to publish, or preparation of the manuscript.

#### Disclaimer.

Authors hold sole responsibility for the views expressed in the manuscript, which may not necessarily reflect the opinion or policy of the *RPSP/PAJPH* and/or PAHO.
